# A new insomnia treatment service: the benefits and challenges of establishing a trainee-led service

**DOI:** 10.1192/bjb.2023.46

**Published:** 2024-04

**Authors:** Lauren Z. Waterman, Michael Creed

**Affiliations:** 1 Health Service and Population Research, South London and Maudsley NHS Foundation Trust, London, UK; 2 Psychosis Studies, Institute of Psychiatry, Psychology & Neuroscience, King's College London, London, UK

**Keywords:** Insomnia, sleep medicine, cognitive–behavioural therapy, innovation, public health

## Abstract

Chronic insomnia is undertreated in the UK despite being a common mental disorder that severely affects quality of life. The lead author, a psychiatry trainee, implemented a new group cognitive–behavioural therapy for insomnia (CBT-I) service for secondary care patients in London with chronic insomnia and comorbid mental illness. Expertise was propagated by trainees teaching other trainees. Nine patients completed all sessions, all with moderate-to-severe insomnia on the Insomnia Severity Index (ISI) at baseline assessment (mean score 21.6). All patients seen at follow-up had improved, scoring in the ‘subthreshold’ or ‘no clinically significant insomnia’ ranges on the ISI (mean 6.6), and all with improvements in comorbid psychiatric symptoms and functioning. This evaluation demonstrates that group CBT-I can be easily learned and delivered by those without formal CBT or sleep medicine training. This could increase the availability and accessibility of treatment. However, bureaucratic challenges were faced, and trainee-led innovations should be better facilitated.

Service development and quality improvement have been central tenets of the UK's National Health Service since the turn of the century.^1^ More recently, the Five Year Forward View for mental health emphasised the importance of responsive, accessible and evidence-based psychotherapies for common mental disorders, improving access for patients from primary care but also for those with more complex mental health needs.^2,3^ This seems particularly relevant to the common mental disorder of chronic insomnia, given its high prevalence in both the general population and those with comorbid mental illness and the dearth of services for its treatment in the UK.

## The rationale for setting up a new insomnia service

Chronic insomnia is a common mental disorder, with an estimated prevalence of 6–39% in the UK.^4^ It is defined as a persistent, subjective disturbance in initiating or maintaining sleep, despite adequate opportunity to, with resulting impact on performance during the day and significant associated distress.^5^ It is shown to severely affect quality of life^6^ and can increase the risk of comorbid mental disorders, reduce efficacy of mental health treatments and precipitate relapse.^7^ Insomnia is, more often than not, comorbid with other mental disorders. This is now known to be due to a bidirectional relationship, with insomnia increasing the risk of a person later developing other mental disorders and *vice versa*.^7^ It is also associated with worse physical health, such as increased risk of mortality from cardiovascular disease, cerebrovascular disease and cancer.^8^

Once chronic, insomnia rarely resolves spontaneously.^9^ Pharmacological treatments have been shown to be effective in insomnia, but there are concerns over risks of tolerance and dependence in some people if prescribed for long-term use.^10^ On the other hand, withdrawing sleeping tablets from people with chronic insomnia, without an effective alternative treatment, would leave them with an untreated long-term condition that significantly affects their health and quality of life.^11^

Sleep hygiene, the most commonly used psychoeducation intervention for insomnia in primary and secondary care, is ineffective for most people with chronic insomnia when used on its own and it was originally intended only for people with mild-to-moderate transient sleep disturbances, rather than chronic insomnia.^12,13^ Conversely, cognitive–behavioural therapy for insomnia (CBT-I) is a specific, manualised form of CBT that combines sleep hygiene with evidence-based behavioural interventions, including stimulus control and sleep restriction.^14^ Studies have consistently shown CBT-I to be the most effective treatment for chronic insomnia, with a high effect size of 0.98 for improving insomnia symptoms^15^ and high cost-effectiveness.^16^ It can be provided with a low burden on resources, is suitable as a group intervention delivered across five sessions^7,17^ and can be successfully delivered by people without specialist CBT training.^17^ In the UK, it has been recommended by the National Institute for Health and Care Excellence (NICE) as the first-line treatment.^18^ As well as providing long-term recovery from insomnia, CBT-I has been shown to prevent and reduce symptoms of many comorbid mental disorders and to improve functioning.^19–21^

Despite its strong evidence base and recommendation as a first-line treatment, CBT-I is not universally available or accessible throughout the UK in primary or secondary care. Insomnia is likely to be particularly prevalent within mental health services, given that insomnia commonly exists as comorbid insomnia. However, in the UK, secondary care mental health services rarely provide CBT-I to their patients.

## Service innovation opportunities for psychiatry trainees

Psychiatry training in the UK is a stepwise, rotation-based system. Trainees typically complete 6 years of psychiatry training, rotating through different psychiatric subspecialties every 6 months.^22^ As in other specialties, trainees learn a significant proportion of their skills through experiential learning, using the model of ‘see one, do one, teach one’. The short time that trainees are within a particular clinical service can be considered a hindrance to innovation and service development, particularly regarding longevity of those innovations. However, this rotational system also offers an opportunity for trainees with a less-common clinical skill set to enter multiple teams and spread that skill set to others in those teams, who might be able to ensure the innovation's longevity, and to other trainees in other teams.

Given the clinical significance of insomnia, the gaps in the provision of its treatment and the potential to increase clinician training and treatment availability, a trainee-run insomnia treatment service was established in south London. In this article, the trainees running that service aim to describe how a trainee-led service was initiated and run, while reflecting on the challenges faced and how some of these were overcome.

## Method

### Clinician training

L.Z.W. obtained informal training at a specialist sleep clinic in London. This consisted of shadowing initial psychiatrist-led insomnia assessments, a five-session CBT-I treatment group and some follow-up review appointments, then leading another five-session CBT-I treatment group from start to finish under direct observation. Further knowledge was obtained by reading the manualised CBT-I guide.^23^

Expertise was spread by other trainees shadowing her, then leading assessments and a group with her direct observation, and then leading a group under indirect supervision. Whenever one trainee was leading a group, another trainee observed that group, then went on to lead their own group, creating a continuous flow of training and learning in order to spread expertise. There was a long waiting-list of trainees keen to shadow a group. So far, four psychiatry trainees have been involved in the programme. Additionally, the programme was shadowed by an assistant psychologist and a mental health MSc student.

### Identifying a suitable patient group

CBT-I is recommended by NICE for patients with chronic insomnia, also known as insomnia disorder, or for those with short-term insomnia that is likely to become chronic. Although there are differing definitions of chronic insomnia, our group was targeted at patients meeting DSM-5 criteria for insomnia disorder.^5^ Patients had to be already under the care of South London and Maudsley NHS Foundation Trust community mental health teams (CMHTs) and meet thresholds for continuing CMHT input.

The manualised guide advises against including patients with a history of mania owing to the risk that the sleep restriction elements of CBT-I will trigger a manic episode. However, there is limited evidence of this occurring. As trainees, we sought to balance the benefits of being more inclusive against the risks of adverse outcomes from working with a higher-risk group. Ultimately, patients with a history of bipolar affective disorder were included provided they did not have a rapid-cycling subtype or recent episodes of mania/hypomania and were continuing to be under a CMHT for ongoing monitoring during the 3 months following treatment. Similarly, patients were deemed unsuitable for the intervention should they be currently experiencing a mental health crisis (such as having had a recent psychiatric hospital admission or being likely to require one imminently).

Owing to additional challenges of running group interventions that include people who have cognitive impairment, drug and alcohol misuse and psychotic beliefs, patients with these conditions were excluded, as per the manualised guide.^23^ Waite and colleagues trialled a CBT-I service for people with psychosis and comorbid insomnia, but this was run as individual therapy to avoid these challenges.^24,25^

### Initiating the service

L.Z.W. approached the trust's CBT lead and proposed the service. The CBT lead agreed to act as a CBT supervisor for governance purposes. As he had no specific experiences of CBT-I, an external CBT-I expert agreed to provide additional advice where needed during the assessment and treatment processes. A brief but detailed service proposal was written and approved by the service manager. The CBT-I service sat within the psychological therapies team that the CBT lead was involved with. Referrals were accepted from two of the trust's boroughs. However, there was no administrative support available, which significantly increased the workload for the trainees running the service. Therefore another team was identified for cohort 2 and the service was included in the team's stepped care pathway, with the team's psychology assistant assigned to support with administration and co-facilitation.

Referrals were initially accepted from the borough covered by that team and one other borough, but the team's service managers noted that more referrals had come from the other borough and this was not to be allowed for future cohorts. Since this was the only CBT-I service available to the trust's patients, the trainees were reluctant to re-run the group only including patients from one borough. Each borough's medical directors were reluctant to accept referrals from different boroughs. Although one borough's management team provisionally agreed to support the service, there were unstated bureaucratic barriers, unclear to the trainees, that meant this never came to fruition. A year later, M.C. commenced a rotation within another psychological therapies team who agreed to house the service for cohort 3.

### Advertising the service and inviting referrals

The mailing lists of all working-age adult CMHTs (other than those solely for patients with psychosis, for reasons explained above) were emailed to inform them of the service, the potential benefits of CBT-I and the inclusion/exclusion criteria. An in-email referral form was included, rather than a lengthy attached form, to reduce the burden of work on referring clinicians.

### The CBT-I intervention

The CBT-I intervention followed the manualised guide by Edinger & Carney for group CBT-I.^23^ The programme consisted of five weekly group therapy sessions, each lasting 1.5 h, as well as an individual assessment and individual follow-up session for each patient. Any suggested medication changes relating to sleep were discussed with the patient and a doctor from their referring CMHT. For the first cohort, initial assessments and the first group session were conduced in person in March 2020, but then moved to virtual via videoconferencing because of the COVID-19 pandemic. The initial assessment covered a detailed biopsychosocial assessment of the patient, with focus on history of sleep difficulty, impact of insomnia on their daytime functioning, interaction of their insomnia with any comorbid mental illnesses or medications, use of sleeping tablets, history of engagement with psychotherapies, screening for any contraindications for treatment and screening for any comorbid sleep disorders that might need further investigation (such as sleep apnoea, parasomnias and periodic limb movements). The group sessions covered psychoeducation about sleep and insomnia, use of a sleep diary, behavioural strategies to target insomnia (including sleep stimulus control, sleep hygiene and relaxation exercises), cognitive restructuring to target the unhelpful cognitions that negatively affect sleep, sleep restriction and relapse prevention. The follow-up session 3 months after the end of treatment assessed the patient's sleep using clinical interview, rating scales and the patient's sleep diaries, targeted any residual concerns or unhelpful cognitions and provided advice regarding any ongoing sleep issues.

### Evaluation of the service

The Insomnia Severity Index (ISI),^26^ Dysfunctional Beliefs and Attitudes About Sleep scale (DBAS)^27^ and Work and Social Adjustment Scale (WSAS)^28^ were administered to assess the severity and impact of insomnia symptoms, and the Clinical Outcomes in Routine Evaluation – Outcome Measure (CORE-OM) was used to evaluate the impact of the treatment on other symptoms of mental illness, such as depression and anxiety.^29^ The ISI, DBAS and CORE-OM were administered during the patients’ initial assessment, at the end of the final group session and at the 3-month follow-up review, and the WSAS was administered at initial assessment and follow-up. The Epworth Sleepiness Scale and STOP-BANG Questionnaire were used at assessment to exclude other sleep disorders that may be contributing to insomnia symptoms.

## Results

The service was given permission to proceed in early 2020. Thus far, three groups have been run to completion in 2020, 2021 and 2022. The first group began in-person in March 2020, but had to be paused and restarted virtually because of restrictions introduced as a result of the COVID-19 pandemic. The second and third groups were virtual throughout.

Across the three cohorts, 30 patients were referred to the service, with 17 commencing the treatment group. Of those who did not commence the treatment, two were excluded prior to assessment as they met exclusion criteria, five did not attend their assessment appointments and four were deemed unsuitable after assessment. Furthermore, two patients were deemed eligible for the treatment group but withdrew prior to the first session.

Of the 17 who commenced the group, nine completed treatment. In the first cohort, three patients were unable to continue because of technological difficulties after the group unexpectedly moved online, while in the second cohort three did not attend after the first session, and two stopped attending after the first session of the third cohort. Of the nine who completed treatment, two were lost to follow-up after not attending their 3-month review.

Patients had a range of comorbid mental illnesses, including depression, anxiety, post-traumatic stress disorder and emotionally unstable personality disorder. All of those who completed the group had moderate or severe insomnia at initial assessment (mean ISA score 21.6; Table 1). All had a reduction in their ISI scores post-treatment (to a mean score of 12.1), with all those seen at follow-up either having absence of or subthreshold insomnia (mean score 6.6). Mean scores on the DBAS reduced from 6.7 pre-treatment to 3.5 post-treatment and 2.5 at follow-up. Mean CORE-OM scores reduced from 2.3 pre-treatment to 1.8 post-treatment and 1.4 at follow-up. Mean scores on the WSAS reduced from 24.1 (severe impairment) pre-treatment to 5.7 (low impairment) at follow-up.

**Table 1:** Outcome rating scale scores for patients who completed group cognitive–behavioural therapy for insomnia (CBT-I)

	Insomnia Severity Index (ISI) score			Dysfunctional Beliefs and Attitudes About Sleep scale (DBAS)			Clinical Outcomes in Routine Evaluation – Outcome Measure (CORE-OM)			Work and Social Adjustment Scale (WSAS)	
	Pre	Post	3-month review	Pre	Post	3-month review	Pre	Post	3-month review	Pre	3-month review
Patient 1	20	16	−	6.1	3.9	−	2.2	2.0	−	18	−
Patient 2	21	14	6	4.6	2.4	1.3	1.9	2.2	1.6	28	5
Patient 3	24	20	12	6.8	5.9	4.5	1.2	1.3	0.7	21	8
Patient 4	21	10	−	5.8	3.4	−	2.4	1.8	−	23	−
Patient 5	21	15	11	7.2	2.1	1.6	2.4	2.8	−	24	12
Patient 6	17	8	2	6.1	3.3	2.3	2.3	1.8	1.3	23	2
Patient 7	25	17	5	9.3	4.8	2.8	2.9	2.2	1.5	28	8
Patient 8	23	8	8	7.9	2.4	2.6	2.5	1.8	2.0	38	5
Patient 9	22	1	2	6.7	2.9	2.4	2.6	0.7	1.3	14	0
Mean Scores	21.6	12.1	6.6	6.7	3.5	2.5	2.3	1.8	1.4	24.1	5.7

Pre, pre-treatment; post, post-treatment.

a. ISI scoring: 0–7 no clinically significant insomnia; 8–14 subthreshold insomnia; 15–21 clinical insomnia (moderate severity); 22–28 clinical insomnia (severe). Changes in ISI score (per Morin et al 2011^26^): ISI reduction of >9.9 indicates marked improvement; reduction of 8.4–9.9 indicates moderate improvement; reduction of 4–8.4 indicates mild improvement.

Both of the patients who had been taking hypnotic medications ceased taking them during treatment and remained off them at follow-up.

## Discussion

As far as the authors are aware, aside from a handful of specialist centres, this is the only doctor-led CBT-I service in the UK. Substantial improvements in outcome measures suggest such services could improve insomnia, as well as comorbid mental illness and overall functioning, in patients under secondary care mental health services, The findings also suggest that new services can feasibly be set up by junior doctors or trainees to improve access to treatment for patients. This could increase the availability and accessibility of an effective treatment which is not widely provided in secondary care in the UK. However, in this case, setting up and running a new service was not without its challenges and pitfalls. Here, we reflect on the clinical outcomes of the service, the challenges that were encountered, how some of these challenges were overcome, how these reflections have influenced the future direction of the service and goals for improving it, and what this learning might mean for other trainees setting up mental health services. While not all the learning would be generalisable to trainees working in other clinical systems within the UK and abroad, these reflections provide important learning points for trainees aiming to instigate a CBT-I service or another type of mental health intervention, and the wider healthcare system.

### Clinical outcomes of CBT-I within a trainee-led service

The outcomes from this service suggest that group CBT-I can be a highly effective treatment for people with chronic insomnia who are under the care of CMHT services. This service was run by psychiatry trainees without formal sleep medicine or CBT qualifications, yet the efficacy of the intervention when followed to completion was broadly similar to the efficacy found in clinical trials and in specialist sleep services in terms of improvement in insomnia symptoms, as well as anxiety and depressive symptoms. This suggests that CBT-I may be easily learned and delivered by psychiatry trainees and probably other mental health professionals with experience in psychological therapies.

There are some limitations to the statistical evaluation of the service presented here. The sample size thus far is small, with only three treatment groups completed, and there was a high attrition rate.

### Challenges encountered

A number of challenges were encountered in setting up the service and within the intervention itself. Some of these were overcome but others have remained and require further consideration.

Bureaucratic barriers may not be shared with trainees and can remain opaque and confusing, which consumes time and energy that would be better used in direct service provision. It appears that multiple bureaucratic layers of management within health services can be an obstacle to innovation, which perhaps particularly affects trainees, who would not routinely be privy to managerial discussions. This challenge needs to be considered not only by trainees but by each layer of management. Rotating trainees can offer fresh ideas and perspectives, without yet being clouded by rigid systems that can absorb those who have remained within a service for longer. Structures should be put in place that encourage and facilitate trainee-led innovations.

There were also organisational barriers to creating a cross-borough service. An underfunded system can create division whereby sectors become protective of their resources, reluctant to share with other sectors. However, where resources, such as the availability of appropriately trained staff, are limited, these can go further if shared. Centralised services should be offered to widen access to important treatments and to not extend an existing postcode lottery by applying divisions within a single trust's coverage.

A limitation of the service was the relatively low number of referrals received in comparison with the estimated rates of chronic insomnia within the secondary care mental health population. When the service was mentioned to care coordinators and team doctors in person, there was strong appetite for it. Therefore, the lower referral rates are likely to have indicated a lack of knowledge about the new service. Advertisement emails can be overlooked in busy clinicians’ inboxes and limited time and resources were available to advertise. There were year-long gaps between groups being run due to the time and resources available to the trainees running them, and bureaucratic delays occurred as previously described – this meant that referrals were often received after groups had commenced or a long time before the next group was due to commence. Should the service become more readily established with more regular groups being run, an increase in referrals is anticipated as these could be received on a continuous basis rather than sporadically.

Additionally, there were challenges in running the group virtually, which appear to have contributed to the high drop-out rate. During the first cohort, digital exclusion was a problem that prevented some patients continuing when the group was forced to suddenly move online. The impact of digital exclusion was not fully clear for the later cohorts, as those who would be unable to attend a virtual group may not have been referred, although some patients struggled with connection problems, which also affected the functioning of the group. Some patients seemed to be ‘half in half out’ during groups, which was difficult to monitor in the virtual environment. Previous research into internet-delivered CBT-I has centred on computerised self-help programmes and individual therapist-led CBT-I, rather than virtual groups;^30^ and although some recent pilot evidence on therapies for other conditions suggests comparable efficacy between online and in-person group therapies for those who complete interventions,^31,32^ completion and attendance rates have been low^32^ or undocumented.^31^ Weinberg has highlighted some of the obstacles inherent in running groups online, such as the therapist's reduced control through body-to-body interaction, and that the online format is thus evidently not for everyone.^33^ This is an emerging area and further research is required.

### Use of the ‘see one, do one, teach one’ model

This service operates with a ‘see one, do one, teach one’ model of learning, allowing trainees new to the service the opportunity to shadow and assist the lead therapist (a trainee familiar with the CBT-I model), before going on to deliver the intervention under appropriate supervision. The recent third cycle of the service was delivered by the second author (M.C.), who assisted with the delivery of the earlier treatment groups. This model could be self-sustaining, allowing trainees to learn and later deliver CBT-I as they progress through training, as well as developing skills at providing brief insomnia interventions in their general clinical practice.

The regular rotation of trainees within and between healthcare trusts was beneficial to this, as it facilitated the spread of knowledge and enabled trainees to make use of new teams in which to house the service. However, there are also challenges associated with the rotational system. Time and energy were needed to repeatedly propose the CBT-I service to new teams, build it into job plans and construct administration arrangements. Additionally, it affects the longevity of the service in any given locality if only rotating trainees are involved.

### Future direction and goals for improvement

This is a nascent service which appears to hold a lot of potential, but further development is needed. Although the outcomes have been positive in those completing treatment, there has been a high attrition rate of 53% of those accepted for treatment, with all disengaging either before or after the first session. As the group has been conducted online, this could be due to people's discomfort with the digital format or with the nature of group treatment. Further evaluation could be conducted by exploring the reasons why patients disengage from treatment to learn how to best adapt the service to meet patients’ needs.

As the service is currently run by trainees, longevity will need to be attained by training more staff in CBT-I, which would ideally include substantive in addition to rotational staff. To expand the pool of staff trained to deliver CBT-I, this could include staff from other clinical backgrounds (e.g. psychologists or community mental health nurses). Benefits of having a CBT-I service led by doctors (or other clinicians with prescribing expertise) were the ability to collaboratively down-titrate sedative medications within the treatment programme as patients’ sleep improved; being able to screen patients for comorbid sleep disorders that might be affecting their sleep, such as sleep apnoea or restless legs syndrome; and understanding how comorbid physical and mental illnesses may be interacting with their insomnia. If the service were to be led in the future by non-medical professionals, alternatives might be to ask patients’ general practitioners to consider down-titration of medication during or after the course of treatment; working alongside a team doctor to discuss options for medication changes; a doctor conducting initial assessments but a non-medical professional leading the group treatment; and/or use of a sleep disorder screening questionnaire during the initial patient assessment, as is done in some services.

Referral and treatment pathways could be further refined, including allowing direct referrals from primary care as well as secondary care. Additionally, the service should be evaluated for health disparities to ensure that the design of the service and referral pathways do not disadvantage some groups. Other outcomes for potential evaluation could be the use of sedative medications, number of hospital admissions or length of CMHT treatment.

We would like to be able to expand the patient group to which this treatment can be offered. Individual CBT-I for patients with insomnia who may not be as suitable for the group intervention could be considered, and research into the use of CBT-I for patients with psychosis, drug and alcohol misuse and cognitive impairment is urgently needed. Furthermore, for patients still unable to access CBT-I or on waiting lists for treatment, clinicians should be trained to offer brief techniques from CBT-I which, in our experience, can be implemented in routine consultations.

Finally, to eliminate the challenges found with running the groups virtually, with the removal of UK COVID-19 restrictions, future groups will be run in-person.

## Data Availability

The full data from rating scales used in this evaluation are presented in this article. Demographic data that may identify individual patients are not available because of the small sample size.

## About the authors

**Lauren Z. Waterman** is a Higher Psychiatric Trainee at South London and Maudsley NHS Foundation Trust, London, and an MD (Res) doctoral student at the Institute of Psychiatry, Psychology & Neuroscience, King's College London, London, UK. **Michael Creed** is a Clinical Fellow at the Institute of Psychiatry, Psychology & Neuroscience, King's College London, London, UK.
